# Investigation of GluA1 and GluA2 AMPA receptor subtype distribution in the hippocampus and anterior cingulate cortex of Long Evans rats during development

**DOI:** 10.1016/j.ibror.2020.03.003

**Published:** 2020-04-03

**Authors:** Nikolaos Tzakis, Matthew R. Holahan

**Affiliations:** Department of Neuroscience, Carleton University, 1125 Colonel by Drive, Ottawa, Ontario, K1S 5B6, Canada

**Keywords:** Hippocampus, ACC, Development, AMPA, GluA1, GluA2

## Abstract

Preadolescent development is characterized by a reorganization of connectivity within and between brain regions that coincides with the emergence of complex behaviors. During the preadolescent period, the rodent hippocampus and regions of the frontal cortex are remodelled as the brain strengthens active connections and eliminates others. In the developing and mature brain, changes in the properties of α-amino-3-hydroxy-5-methyl-4-isoxazolepropionic acid receptors (AMPAr)-mediated synaptic responses contribute to experience-dependent changes in neural organization and function. AMPAr are made up of 4 subunits, of which GluA1 and GluA2 have been shown to play the most prominent role in functional plasticity. In this study, we sought to determine whether levels of these two subunits changed during the course of pre-adolescent development in the hippocampus and anterior cingulate cortex (ACC). To investigate the developmental changes in GluA1 and GluA2 AMPAr subunits, Western blotting and immunohistochemistry were performed on the ACC and hippocampus from P18 - P30 and compared to adult (P50) levels and distribution. Within the hippocampus, protein levels of GluA1 and GluA2 peaked around P26−30 whereby localized staining in the dentate gyrus reflected this pattern. GluA1 and GluA2 levels within the ACC showed little variation during this developmental period. These results indicate that changes in AMPAr subunits within the hippocampus coincide with developmental modifications that underlie the shift from juvenile- to adult-like capabilities. However, changes in AMPAr distribution in the ACC might not mediate changes that reflect preadolescent developmental shifts.

## 1 Introduction

During neural development, a high degree of pruning and remodelling is required for the brain to achieve its final, fully functioning adult configuration (Kantor and Kolodkin, 2003). These connectivity-based changes occur during sensitive periods that can be brain-wide or in localized regions (Fenoglio et al., 2006) and are determined by a combination of innate genetic factors and responses to external stimuli (Sur and Rubenstein, 2005). During the preadolescent period (ending approximately at postnatal (P) days 32–39 in rats; Dutta and Sengupta, 2016) neural regions that subserve memory functions, like the hippocampus and frontal cortex, experience developmentally-linked changes in synaptic organization prior to the onset of adulthood. These maturational changes are likely dependent on fluctuations in synaptic excitation, mediated by glutamatergic mechanisms, and synaptic inhibition, mediated by GABAergic mechanisms (see Jensen, 2002 Fig. 4 for summary).

Within the hippocampus, neurogenesis in the dentate gyrus reportedly peaks in density between P15 and P18 (Altman and Das, 1965; Bayer, 1980; Zhao et al., 2006; Curlik et al., 2014). Mossy fibers, which form connections between hippocampal granule cells in the dentate gyrus and CA3 pyramidal cells, extend from the stratum lucidum to the stratum oriens of the CA3 from P18 to P24 where they remain stable into adulthood (Amaral and Dent, 1981; Gaarskjaer, 1985, 1986; Holahan et al., 2006, 2007). This phase of hippocampal modification is hypothesized to underlie a “switch” from juvenile- to adult-like functioning (Holahan et al., 2019).

The prefrontal cortex is thought to undergo a series of remodeling and structural modifications that occur slightly later in development than the hippocampus. The colocalization of astrocytes with dendritic markers are reduced between P24 to P30, while markers of pre- and post-synaptic maturation show prolonged development that reach adult like levels around P36 for the pre-synaptic component (starting around P29) and P30 for the post-synaptic compartment (starting around P22; Pinto et al., 2013; Testen et al., 2019). An RNA-sequencing study of the rat frontal cortex revealed a change in gene expression patterns from regulation of network formation to maintenance around P21 (Kroeze et al., 2018). The authors suggested that these changes in gene expression may contribute to the formation of mature frontal cortical circuits that underlie stability of cognitive processes (Kroeze et al., 2018).

Input-dependent changes in synaptic organization, such as those that occur during development, have been shown to be dependent on the distribution and/or composition of post-synaptic α-amino-3-hydroxy-5-methyl-4-isoxazoleproprionate receptors (AMPAr), including changes in their binding affinity, number of binding sites, or a change in their ion channel kinetics (Maren et al., 1993; Henley et al., 2011; Lisman et al., 2012). AMPAr are concentrated at synapses where they mediate fast excitatory post-synaptic currents. They are composed of a combination of four subunits, GluA1-4, with each combination of subunits dictating the distinct functional properties of the receptor (Hollmann and Heinemann, 1994; Schoepfer et al., 1994; Dingledine et al., 1999; Murphy et al., 2012; Henley and Wilkinson, 2016). The majority (∼80 %) of synaptic AMPAr in CA1 hippocampal neurons are composed of GluA1 and GluA2 heteromers, while subunits containing GluA3 occur at much lower rate (∼10 %). GluA4 is developmentally regulated, with expression peaking during early postnatal development and decreasing thereafter, such that glutamatergic synapses in principal neurons in adult brains sparsely express the GluA4 subunit (Henley and Wilkinson, 2016). In fact, GluA4-containing AMPAr delivered into synapses following spontaneous neural activity are reportedly exchanged with non-synaptic GluA2-containing AMPAr (Zhu et al., 2000). The remaining GluA4-containing AMPAr play a role in mediating transmission in interneurons, especially parvalbumin-containing inhibitory interneurons (Henley and Wilkinson, 2016).

GluA1 and GluA2 expression vary considerably throughout development. In the frontal cortex, levels of AMPAr (no specific subunit) show a steady increase from P0 – P20 at which point they declined to adult levels (Insel et al., 1990). Hippocampal levels show a similar pattern of expression with the CA1 region showing higher expression than the CA3 or dentate gyrus (Insel et al., 1990) but all regions attained adult levels of AMPAr expression by P30. mRNA expression of the GluA1 subunit showed a two-fold increase from P1 to P28 in the hippocampus but no change in the frontal cortex (Durand And Zukin, 1993) while GluA2 mRNA expression showed a six-fold increase in expression in the hippocampus from P1 to P28 and a two-fold change in the frontal cortex (Durand and Zukin, 1993). In the cortex, immunohistochemical labeling of GluA1 peaked between P10 and P20 (100 % increase from embryonic day (ED) 14 to P10) then decreased by 40 % to adult levels (Arai et al., 1997). GluA2-positive neurons showed a 40 % increase from ED18 to P10 then declined by approximately 10 % to adult levels (Arai et al., 1997). In the hippocampal CA3 region, GluA1- and GluA2-positive neurons increased by 100 % from ED18 to P0 and remained at that level to adulthood (P50; Arai et al., 1997). In another report (Martin, et al., 1998), GluA1-positive neurons achieved their adult pattern after P21 and in the hippocampal CA1 region, maximal levels of GluA1 expression were noted at P19 with a subsequent 15–20% decline by P21.

Hippocampal GluA2 expression is low relative to GluA1 expression around postnatal (P) days 3–5, consistent with prior reports that attribute GluA2-lacking AMPAr to neonatal synaptic function (Pickard et al., 2000; Henley and Wilkinson, 2016). Expression of GluA1 within the hippocampus continues to increase between P5 and P21, after which there is a shift whereby GluA1 expression levels decline and GluA3 levels increase (Pellegrini-Giampietro et al., 1992b; Pickard et al., 2000; Henley and Wilkinson, 2016). This results in a reduction in the threshold for postsynaptic LTP induction and concurrent increase in the presynaptic LTP induction threshold (Dumas, 2012; Blair et al., 2013). By P14, almost all AMPAr synapses express GluA2, suggesting a developmental “switch” from Ca^+2^-permeable to -impermeable AMPAr during early development (Pellegrini-Giampietro et al., 1992b; Pickard et al., 2000; Henley and Wilkinson, 2016).

In the current investigation, Western blotting was performed to evaluate the variation in expression levels of GluA1 and GluA2 AMPAr subunits within the hippocampus and ACC from P18 - 30 and compare them to adult (P50) levels. Immunohistochemical analyses were also performed on the hippocampus to localize GluA1 and GluA2 levels during development. The developmental period of P18 – P24 coincides with pre- and post-synaptic modifications within the hippocampus so we hypothesized that changes in GluA1 or GluA2 levels would parallel these connectivity changes. Because frontal cortical circuits, including the anterior cingulate cortex (ACC), show a somewhat more protracted developmental trajectory, we hypothesized that levels of GluA1 and GluA2 in the ACC would occur slightly later from P24 – P30.

## 2 Materials and methods

### 2.1 Subjects

Male Long-Evans rats (LERs) were used throughout the experiment. Rats were born in the temperature and humidity-regulated animal facility at Carleton University. The day the pups were born was marked as P0. Pups were weaned on P18. Newly weaned rats were provided with solid food pellets soaked with some water and placed in a ceramic dish in the cage, along with a dish containing fortified water gel. Dry food pellets were also placed in the cage. For all experiments, rats were group-housed in polycarbonate cages with a 12 -h light-dark cycle: lights on 8h00, lights off 20h00 with food and water provided *ad libitum*. Animal care was conducted in accordance with the Canadian Council on Animal Care (CCAC) guidelines and approved by the Carleton University Animal Care Committee.

### 2.2 Western blotting

Rats were injected with 3 mL urethane at one of seven ages (P18, P20, P22, P24, P26, P30, P50). When they were no longer responsive to a toe pinch, rats were decapitated, and brains were removed from the skull and hemisected. Tissue punches were collected from the ACC and hippocampus from one hemisphere and used for Western blotting quantification for GluA1 (ACC: P18: n = 3; P20: n = 6; P22: n = 5; P24: n = 5; P26: n = 5; P30: n = 5; P50: n = 5; HPC: P18: n = 5; P20: n = 4; P22: n = 5; P24: n = 5; P26: n = 5; P30: n = 5; P50: n = 5) and GluA2 (ACC: P18: n = 3; P20; n = 4; P22: n = 3; P24: n = 5; P26: n = 5; P30: n = 5; P50: n = 5; HPC: P18: n = 4; P20: n = 4; P22: n = 5; P24: n = 4; P26: n = 5; P30: n = 5; P50: n = 5). The other hemisphere was immersion-fixed in 4% paraformaldehyde. Hemispheres were counterbalanced for the two analyses.

Whole cell lysates from the hippocampus were homogenized in Radio Immuno Precipitation Assay (RIPA) buffer [50 mM Tris (pH 8.0), 150 mM sodium chloride, 0.1 % sodium dodecyl sulphate (SDS), 0.5 % sodium deoxycholate and 1% Triton X-100] mixed with 1 tablet of Complete Mini ethylenediaminetetraacetic acid (EDTA)-free protease inhibitor (Roche Diagnostics, Laval, QC, Cat #11 836 170 001) per 10 mL of buffer then sonicated for 10 s in ice cold water. The lysed cells were centrifuged at 5000 RPM for 10 min at 4 °C. The supernatant was extracted, and protein concentration was determined using bicinchoninic acid (BCA) method (Thermo Scientific). Following protein concentration determination, the supernatant was placed in 5x loading buffer (containing 5% glycerol, 5% β-mercaptoethanol, 3% SDS and 0.05 % bromophenol blue) and the protein was denatured by placement in a 5-minute heating block at a temperature of 105 °C. Following this step, samples were stored at -20 °C.

Proteins were separated using sodium dodecyl sulphate-polyacrylamide gel electrophoresis (SDS-PAGE). The SDS-PAGE gel (7.5 %) containing the separating buffer (370 mM Tris-base (pH 8.8), 3.5 mM SDS) and the stacking buffer (124 mM Tris-base (pH 6.8), 3.5 mM SDS) were placed in running buffer (25 mM Tris-base, 190 mM glycine, 3.5 mM SDS) and samples, along with the Precision Plus ProteinTM Standards Dual Color (Bio-Rad, Hercules, CA, Cat#161−0374), were loaded into the Arcylamide gel (7.5 %) for molecular weight determination at 140 V. After electrophoresis, proteins were transferred for one hour at 4 °C at 100 V in transfer buffer solution (25 mM Tris-base, 192 mM Glycine, 20 % methanol) onto a polyvinylidene difluoride (PVDF) membrane (Bio-Rad, Cat# 162−0177). Thereafter, membranes were dried overnight. The following day, membranes were reactivated using methanol and total protein load concentration was determined.

After a brief methanol rinse (5 s), membranes were incubated in REVERT total protein solution for a period of 5 min followed by placement into a REVERT wash solution (6.7 % Glacial Acetic Acid, 30 % Methanol, in water) two times 2 min each. Membranes were quickly rinsed with distilled water and imaged on a LI−COR Odyssey imaging system on the 700 channel for an exposure period of 2 min. Membranes were then rinsed immediately post imaging in tris-buffered saline [(TBS) pH 7.5 (2 × 5 min.)] followed by a block with 0.5 % fish gelatin (Sigma) in TBS for 60 min. Membranes were incubated with rabbit anti-GluA1 (1:1000; EMD Millipore, Cat# AB1504) or rabbit anti-GluA2 (1:4000; EMD Millipore, Cat# AB1768-I) overnight in 0.05 % fish gelatin in TBS with 0.1 % tween. Membranes were washed with 15 mL of TBS-T/membrane at room temperature three times five minutes each. Membranes were then incubated for one hour in infrared conjugate secondary (AlexaFluor 680 goat anti-rabbit; Invitrogen, Cat# AB175773) at a concentration of 1:4000 in 0.5 % fish gelatin solution containing 0.2 % tween and 0.01 % SDS. Membranes were then washed in TBS-T (1 X 5 min) followed by 2 X 5 min washes in TBS and read on a Licor Odyssey system on the 700-channel for 6 min.

Total protein expression from each sample on each membrane was determined using the total protein stain method in order to provide normalization across each specific membrane. To determine protein expression, bands at the appropriate molecular weight were determined with the Odyssey software. Presented data represents the ratio of target protein signal strength to normalized total protein signal strength.

### 2.3 Immunohistochemistry

The hemisphere not used for Western blot analysis was immersion-fixed in 4% paraformaldehyde in 0.1 M phosphate-buffered saline (PBS) overnight at 4℃. This solution was replaced with 30 % sucrose in 0.1 M PBS the following day and brains were stored at 4℃ until sectioning. Brains were coronally sectioned through the ACC and the dorsal hippocampus at 60 μm on a Leica CM1900 cryostat (Weztler, Germany). Sections were stored in a 0.1 % sodium azide solution in 0.1 M phosphate buffer (PB) at 4℃.

For GluA1 and GluA2 immunohistochemistry, sections were placed in phosphate-buffered saline with 0.1 % Triton X (PBS-TX) for three, 5-minute washes. They were then incubated in 0.3 % hydrogen peroxide (H_2_O_2_) in PBS-TX for 30 min, followed by three, five-minute washes in PBS-TX. Sections were transferred to 1x animal free blocker (AFB; Vector) in PBS-TX for 30 min at room temperature. Incubation in the primary antibody anti-GluA1 (rabbit; 1:1000; EMD Millipore, Cat# AB1504) or anti-GluA2 (rabbit; 1:4000; EMD Millipore, Cat# AB1768-I) occurred for 24 h at room temperature. The tissue was washed in PBS-TX for three, 10-minute washes followed by a 2 -h incubation in the secondary antibody (anti-rabbit biotinylated from Vector Laboratories, 1:500, Cat# BA-9200). Tissue was washed for three, 10-minute washes in PBS-TX before being placed into an ABC solution (Vector) for one hour. The tissue was rinsed in three, 5-minute washes using PBS before being placed into a DAB solution. Sections were then mounted on glass slides, dehydrated, and cover slipped. ∼2−3 tissue sections were counted from each brain for P18 (n = 4), P20 (n = 3), P22 (n = 3), P24 (n = 4), P26 (n = 4), P30 (n = 4), and P50 (n = 4) for GluA1 and P18 (n = 3), P20 (n = 4), P22 (n = 6), P24 (n = 4), P26 (n = 3), P30 (n = 4), and P50 (n = 4) for GluA2.

Utilizing unbiased stereological principles, an estimate of the number of GluA1 and GluA2-positive neurons within the dentate gyrus region of the dorsal hippocampus was undertaken. Stained sections were visualized using an Olympus BX51 brightfield microscope with a motorized stage (Olympus Canada, Markham, ON) and images captured with an Olympus U-CMAD3 camera. The region of interest (dentate gyrus) for each section was traced and counted digitally at 40× magnification. Around two to four random coronal sections were sampled from each rat. A minimum of 3 animals per group was included in analysis. Not all animals could be included in analysis due to various factors such as missing sections for some animals. Quantification is represented as an estimated total per mean measured thickness per 100,000 μm^3^ to allow for comparisons across brain sections.

### 2.4 Statistical analyses

For Western blotting and immunohistochemical counts, a one-way ANOVA was run with age of the rat as the independent variable and protein signal strength (Western blotting) or cell count (immunohistochemistry) as the dependent variable. Comparisons between each age groups were conducted using Tukey test post hoc analysis when a main effect of age was present. Data are graphically represented as mean + SEM.

## 3 Results

### 3.1 Western blotting

#### 3.1.1 GluA1

**Fig. 1:**
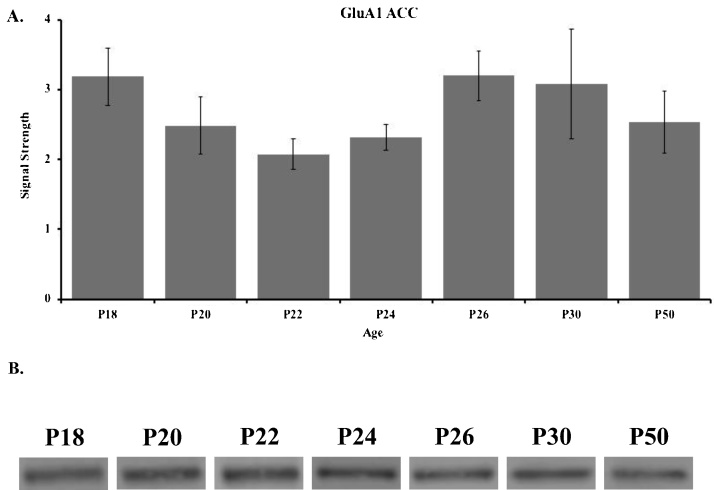
(**A**) Western blot analyses and (**B**) representative bands of GluA1 in the ACC of rats in the P18 (n = 3), P20 (n = 6), P22 (n = 5), P24 (n = 5), P26 (n = 5), P30 (n = 5), and P50 (n = 5) age groups. Data are presented as mean + SEM.

Western blot results for the GluA1 subunit in the ACC are shown in Fig. 1 for P18 (n = 3), P20 (n = 6), P22 (n = 5), P24 (n = 5), P26 (n = 5), P30 (n = 5), and P50 (n = 5). These data show that GluA1 expression in the ACC remained relatively consistent throughout the developmental ages, with a slight decrease at P22 and P24 (Fig. 1, Supplementary Fig. 1A). A one-way ANOVA revealed no significant difference in ACC GluA1 levels across the seven age groups (*F*_(6, 27)_ = 0.977, *p* =  0.482).

**Fig. 2:**
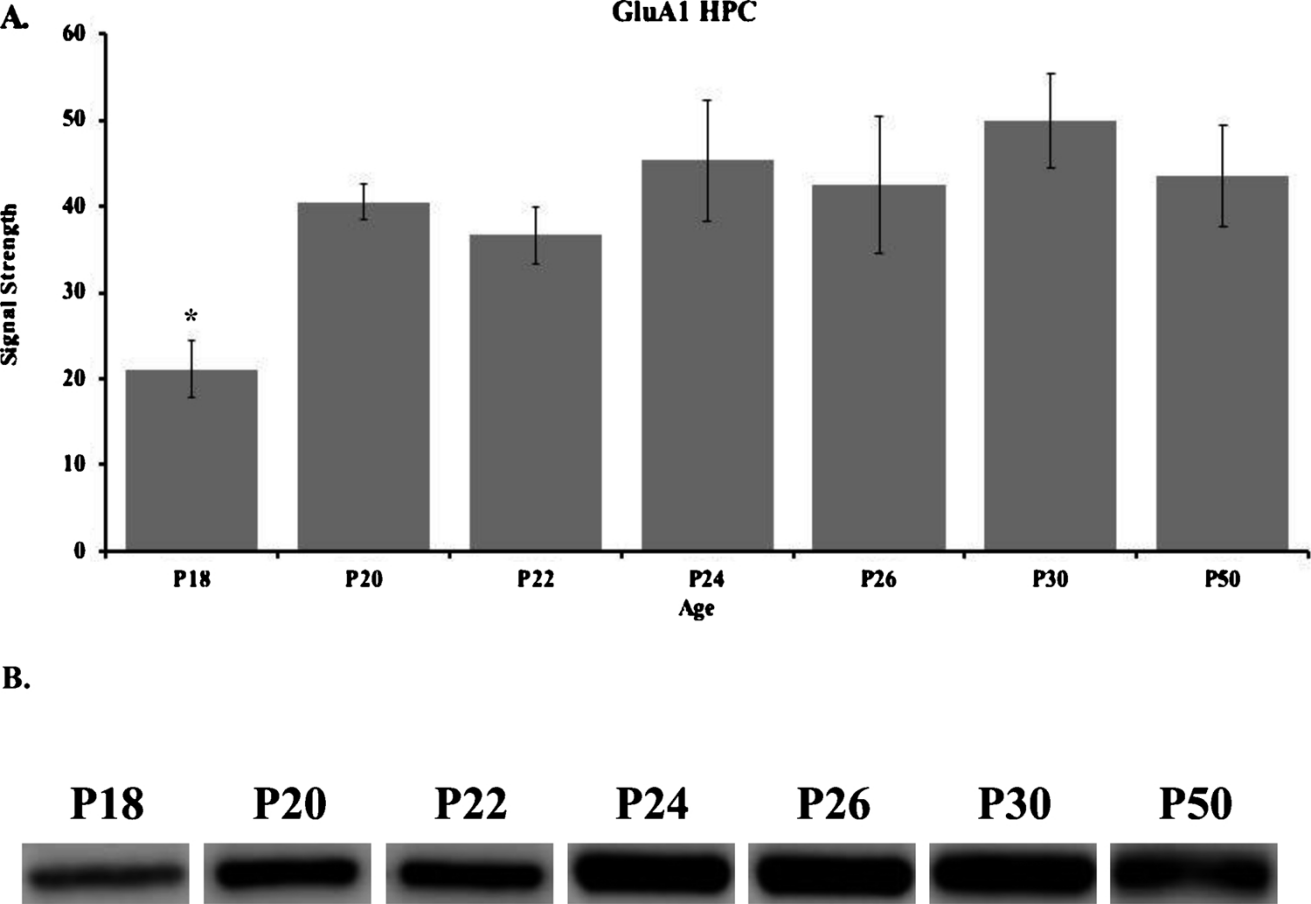
(**A**) Western blot analyses and (**B**) representative bands of GluA1in the hippocampus of rats in the P18 (n = 5), P20 (n = 4), P22 (n = 5), P24 (n = 5), P26 (n = 5), P30 (n = 5), and P50 (n = 5) age groups. (*) indicates significant differences between P18 and P30 groups (*p* < 0.05). Data are presented as mean + SEM.

Western blot results for the GluA1 subunit in the HPC are shown in Fig. 2 for P18 (n = 5), P20 (n = 4), P22 (n = 5), P24 (n = 5), P26 (n = 5), P30 (n = 5), and P50 (n = 5). These data show that GluA1 levels in the hippocampus sharply increased from P18 to 20, and then gradually increased and peaked at P30 and remained stable into adolescence (Fig. 2, Supplementary Fig. 1A). A one-way ANOVA revealed a main effect of age (*F*_(6, 27)_ = 2.890, *p* <  0.05). Tukey post-hoc tests revealed that hippocampal GluA1 levels at P30 were significantly higher than at P18 (*p* < 0.05).

#### 3.1.2 GluA2

**Fig. 3:**
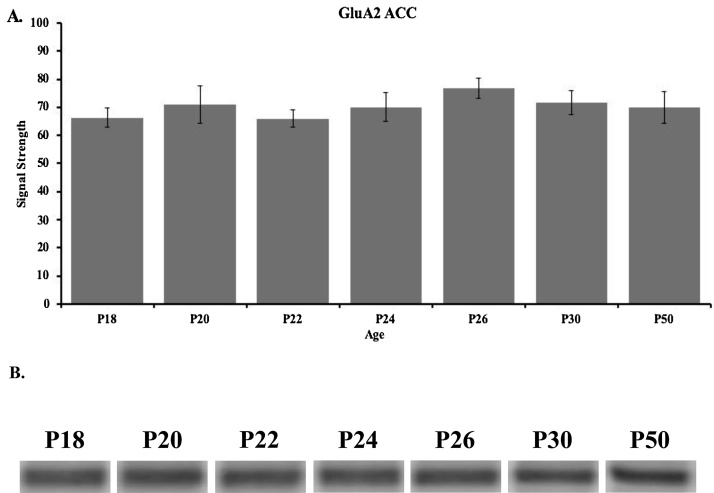
(**A**) Western blot analyses and (**B**) representative bands of GluA2 expression in the ACC of rats in the P18 (n = 3), P20 (n = 4), P22 (n = 3), P24 (n = 5), P26 (n = 5), P30 (n = 5), and P50 (n = 5) age groups. Data are presented as mean + SEM.

Western blot results for the GluA2 subunit in the ACC are shown in Fig. 3 for P18 (n = 3), P20 (n = 4), P22 (n = 3), P24 (n = 5), P26 (n = 5), P30 (n = 5), and P50 (n = 5). These data show that GluA2 levels in the ACC remained consistent throughout the developmental ages (Fig. 3, Supplementary Fig. 1B). A one-way ANOVA revealed no significant difference in ACC GluA2 across the seven age groups (*F*_(6, 23)_ = 0.518, *p* =  0.789).

**Fig. 4:**
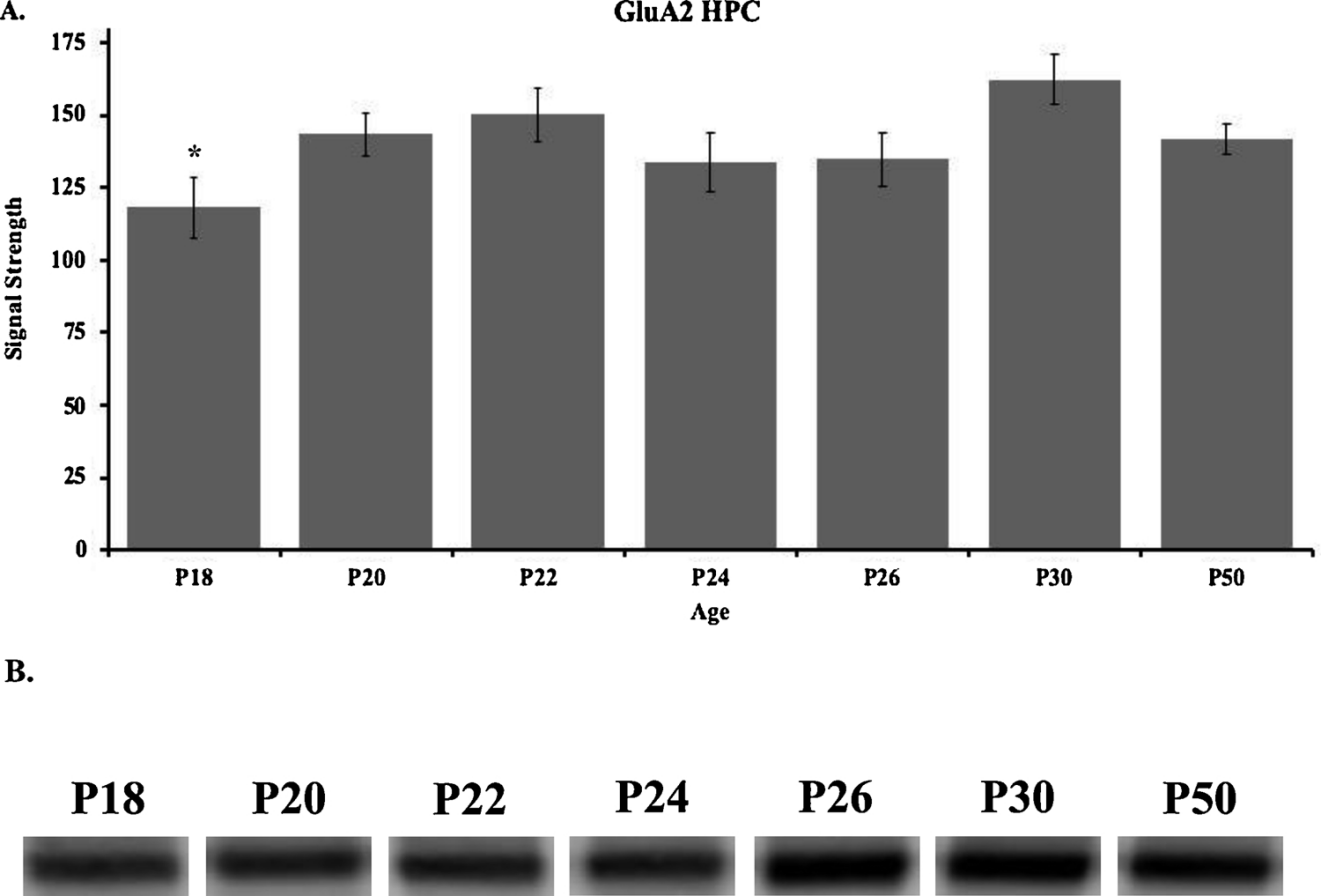
(**A**) Western blot analyses and (**B**) representative bands of GluA2 expression in the hippocampus of rats in the P18 (n = 3), P20 (n = 4), P22 (n = 3), P24 (n = 5), P26 (n = 5), P30 (n = 5), and P50 (n = 5) age groups. (*) indicates significant differences between P18 and P30 groups (*p* < 0.05). Data are presented as mean + SEM.

Western blot results for the GluA2 subunit in the HPC are shown in Fig. 4 for P18 (n = 4), P20 (n = 4), P22 (n = 5), P24 (n = 4), P26 (n = 5), P30 (n = 5), and P50 (n = 5). These data show that GluA2 levels in the hippocampus gradually increased throughout development, peaking at P30 and remaining stable into adolescence (Fig. 4, Supplementary Fig. 1B). A one-way ANOVA revealed a significant main effect of age (*F*_(6, 25)_ = 2.825, *p* <  0.05). Tukey post-hoc tests revealed that hippocampal GluA2 levels at P30 were significantly higher than at P18 (*p* < 0.05).

### 3.2 Immunohistochemistry

**Fig. 5:**
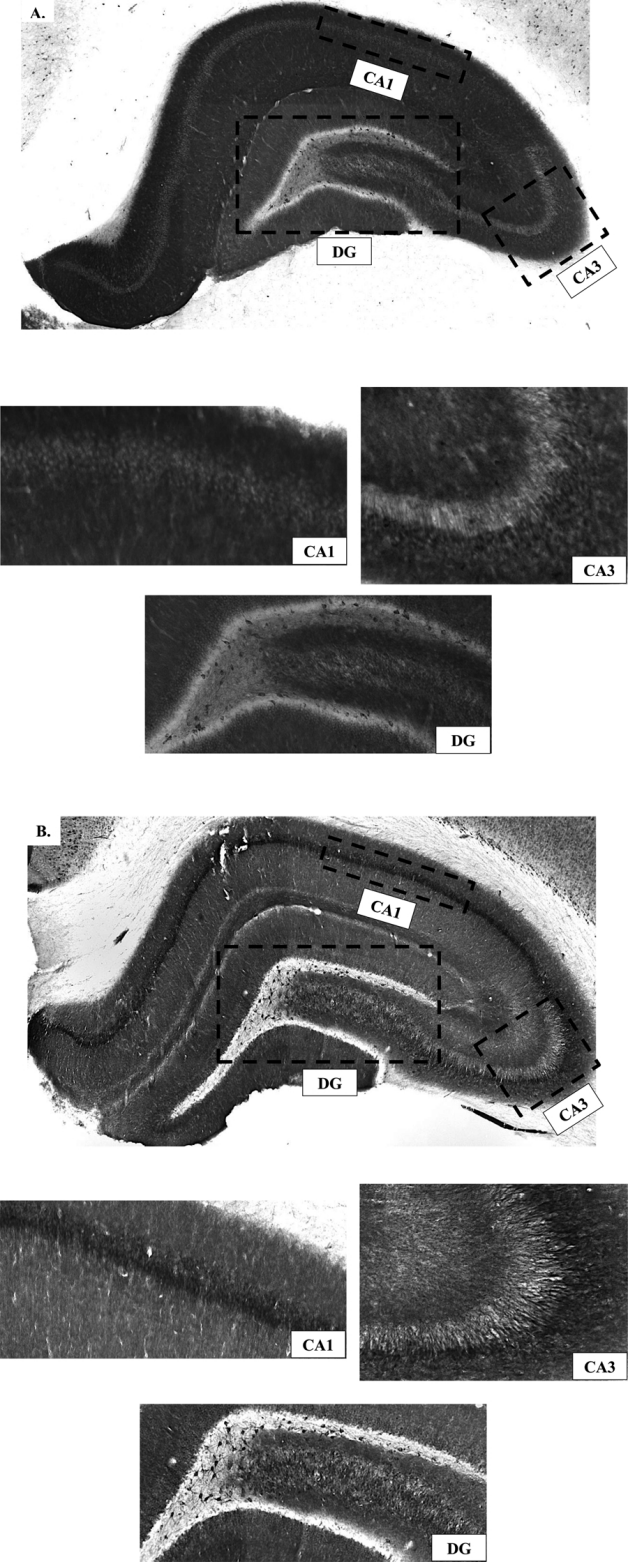
Representative staining within the hippocampus, and CA1, CA3, and dentate gyrus (DG) subregions for (**A**) GluA1 and (**B**) GluA2. Dense staining within the CA1 and CA3 proved to be difficult to provide an accurate count within those regions. As such, counts were localized within the dentate gyrus area of the hippocampus.

Dense staining within the CA1 and CA3 proved to be difficult to provide an accurate count within those regions (Fig. 5). As such, counts were localized exclusively within the dentate gyrus area of the hippocampus (Fig. 5) which showed punctate cellular staining.

**Fig. 6:**
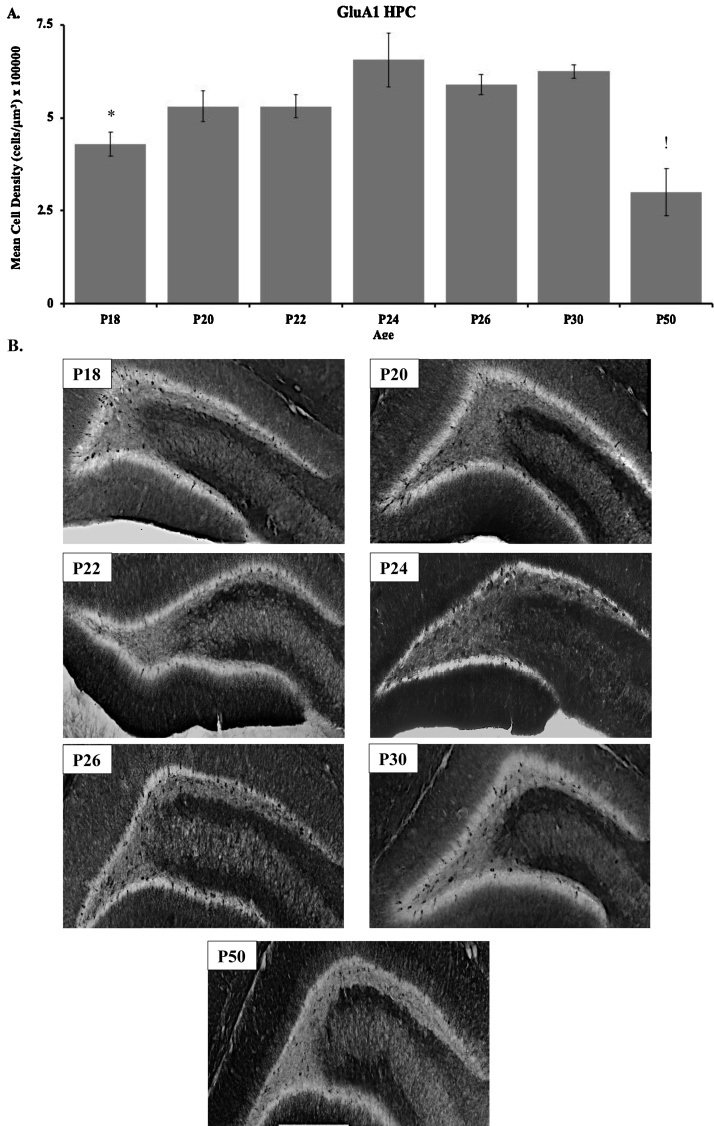
(**A**) Immunohistochemical analyses and (**B**) representative staining of GluA1 within the dentate gyrus of the hippocampus in the P18 (n = 4), P20 (n = 3), P22 (n = 3), P24 (n = 4), P26 (n = 4), P30 (n = 4), and P50 (n = 4) age groups. (*) indicates significant difference between P18 and P24 (*p* < 0.05). (!) indicates significant difference between the P50 group and all other age groups (*p* < 0.05). Data are presented as mean + SEM.

Immunohistochemical results for the GluA1 subunit in the HPC are shown in Fig. 6 for P18 (n = 4), P20 (n = 3), P22 (n = 3), P24 (n = 4), P26 (n = 4), P30 (n = 4), and P50 (n = 4). These data show that GluA1-stained cells in the dentate gyrus increased throughout development and stabilized into adulthood by P24 (Fig. 6). A one-way ANOVA revealed a significant main effect of age (*F*_(6, 19)_ = 7.582, *p* <  0.001). Tukey post-hoc test revealed that dentate GluA1 staining at P24 was significantly higher than P18 (*p* < 0.05). P50 were significantly lower compared to all P20, 22, 24, 26, and 30 (*p* < 0.05).

**Fig. 7:**
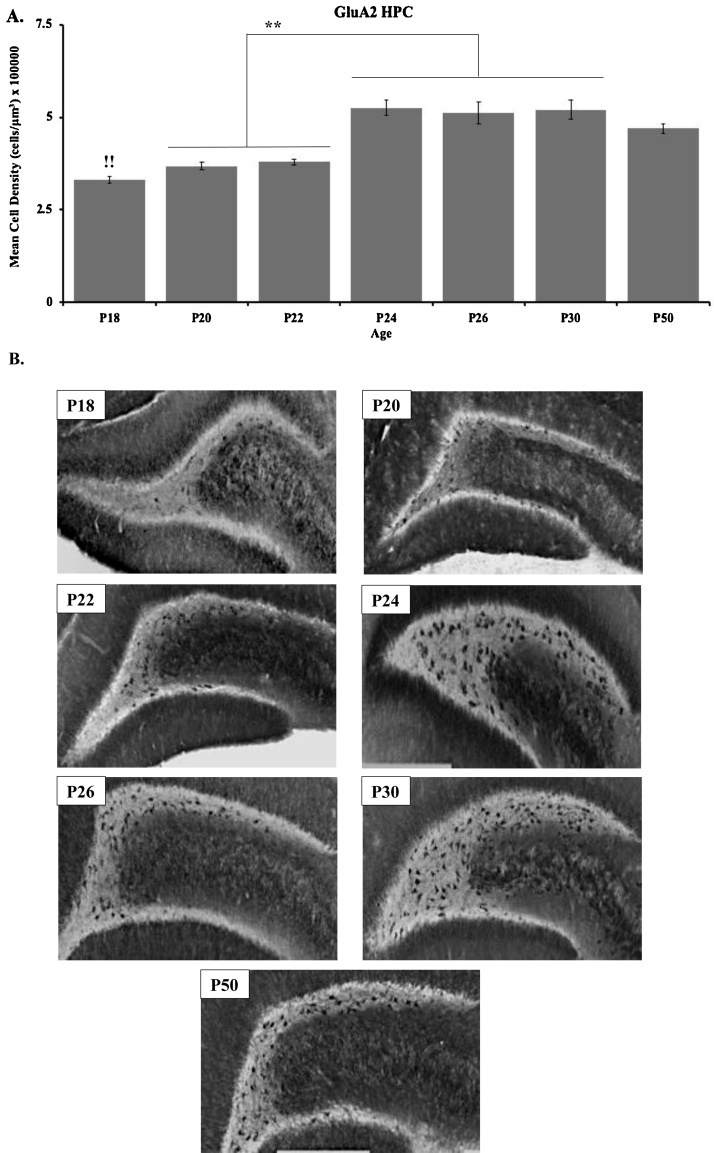
(**A**) Immunohistochemical analyses and (**B**) representative staining of GluA2 within the dentate gyrus of the hippocampus in the P18 (n = 3), P20 (n = 4), P22 (n = 6), P24 (n = 4), P26 (n = 3), P30 (n = 4), and P50 (n = 4) age groups. (!!) indicates significant differences between the P24, 26, 30, and 50 groups and the P18 group. (**) indicates significant differences between the P24, 26, and 30 groups and the P20 and 22 groups (*p* < 0.001). Data are presented as mean + SEM.

Immunohistochemical results for the GluA2 subunit in the HPC are shown in Fig. 7 for P18 (n = 3), P20 (n = 4), P22 (n = 6), P24 (n = 4), P26 (n = 3), P30 (n = 4), and P50 (n = 4). These data show that GluA2 staining in the dentate increased throughout development and stabilized into adulthood by P24 (Fig. 7). A one-way ANOVA revealed a significant main effect of age (*F*_(6, 21)_ = 12.961, *p* <  0.001). Tukey post-hoc test revealed that dentate GluA2 staining at P24, 26, 30, and 50 were significantly higher than P18, and at P24, 26, and 30 compared to P20 and 22 (*p* < 0.01).

## 4 Discussion

The current investigation evaluated developmental changes in levels of GluA1 and GluA2 AMPAr subunits within the hippocampus and ACC from P18 - 30 and compared them to adult (P50) levels. This developmental period coincides with preadolscent connectivity changes in the hippocampus so we hypothesized that changes in GluA1 or GluA2 would parallel these developmental changes. We hypothesized that levels of GluA1 and GluA2 in the anterior cingulate cortex (ACC) would follow a similar pattern but perhaps peak slightly later. Consistent with the first hypothesis, GluA1 levels increased from P18 to P30 in the hippocampus. GluA1 protein level changes across development were reflected in immunohistochemical localization to the dentate gyrus. GluA2 hippocampal levels showed a more linear increase from P18 to 30 while immunohistochemical staining in the dentate revealed an abrupt increase from P22 to P24. Levels of GluA1 and GluA2 in the ACC showed little variation across the developmental timepoints investigated.

Hippocampal GluA1 levels evaluated during postnatal development dramatically increased up to P21, with an expression pattern thereafter similar to that found in the adult (Durand and Zukin, 1993; Ryo et al., 1993; Martin et al., 1998; Ibaraki et al., 1999; Hagihara et al., 2011). A gradual increase in hippocampal GluA1 expression has been reported across P0, 7, 14, and 30, with the highest levels detected at P30 (Bian et al., 2012). Sans et al. (2000) reported a similar developmental increase in GluA1 levels between P2 and P10 and P10 and P35, which remained stable into adulthood. A detailed timeline of changes in GluA1 levels during pre-adolescent development shows a peak sometime after P18, where levels then stabilize into adulthood. This developmental timeline coincides with a period of hippocampal remodeling (reviewed in Holahan et al., 2019), indicating that GluA1 levels may somehow contribute to this remodeling and facilitate the transition from juvenile to adult-like cognitive functions.

We noted a distinct pattern of GluA1 staining on cells within the dentate gyrus that more or less paralleled the overall protein levels in the hippocampus. The regional expression of GluA1 within the hippocampus has been reported to vary dramatically during postnatal development. From P0 to P5, GluA1 is enriched in apical dendrites and the soma of pyramidal cells in the CA2, CA3, and subiculum while the dentate gyrus and CA1 pyramidal cells show low GluA1 expression (Ryo et al., 1993). By P14, GluA1 levels remain elevated in the CA2 and CA3 regions with increasing levels in the CA1, the dentate gyrus, and the subiculum/entorhinal cortex (Ibaraki et al., 1999). After P21, GluA1 levels were highest in the CA1, followed by the dentate gyrus, the CA2 and CA3 regions (Martin et al., 1998; Palomero-Gallagher et al., 2003). The strata of the CA1 also show differential distribution of GluA1 with the stratum oriens labeling emerging before the stratum radiatum (Ryo et al., 1993; Martin et al., 1998). This aligns with the later maturation of the dendritic system, including the branching of apical dendrite lateral fibers into the stratum radiatum and preterminal fibers branching in the stratum lacunosum between P15 and P24. These morphological events coincide with the development of synaptic transmission within the CA1 during which time LTP within this region increases substantially (Pokorný and Yamamoto, 1981; Ryo et al., 1993; Martin et al., 1998).

In the present work, GluA1 levels were significantly lower at P50 than P30. This is in line with previous investigations reporting a similar peak in GluA1 during early development, followed by a decrease into adulthood (Pellegrini-Giampietro et al., 1991, 1992a; Durand and Zukin, 1993; Martin et al., 1998; Ibaraki et al., 1999; Bian et al., 2012; Blair et al., 2013; McHail and Dumas, 2015). After P21, GluA3 expression overtakes and replaces GluA1 expression, which may serve to explain the decrease in GluA1 levels observed in adults (Durand and Zukin, 1993; Martin et al., 1998; Blair et al., 2013; McHail and Dumas, 2015).

Levels of GluA2 are low compared to GluA1 during postnatal development, but high levels have been noted up to P7 (Monyer et al., 1991; Wisden and Seeburg, 1993; Isaac et al., 2007). The developmental trajectory of GluA2 has shown an incremental increase from P0 to P15 with adult-levels reached by P35 (Sans et al., 2000; Pandey et al., 2015). Results from our study indicate that GluA2 protein reached adult levels around P26-30. Other reports have shown that almost 96 % of AMPAr contained the GluA2 subunit by P20 (Pickard et al. (2000). Assessment of GluA2 mRNA expression showed peak levels around P14 (Pellegrini-Giampietro et al. (1991) and Durand and Zukin (1993)), and it is possible that protein reaches adult levels shortly after mRNA peaks, following a series of post-translational modifications (Liu et al., 2016).

Between P7 and P35, GluA2 levels increase in the dentate gyrus and are almost exclusively expressed in mature granule cells (Standley et al., 1995; Hagihara et al., 2011). GluA2 levels within the CA1 and CA3 are slightly lower relative to the dentate gyrus from P0 to P21, after which they begin to increase, reaching similar levels as the dentate gyrus by adulthood (Pellegrini-Giampietro et al., 1992a; Hagihara et al., 2011). Within the CA3, GluA2 subunits are localized to postsynaptic densities on thorny excrescences within the stratum lucidum and on distal dendritic spines and shafts within the strata radiatum and lacunosum moleculare. This distribution suggests that both the excitatory mossy fiber input to CA3 and the associational/commissural input to the stratum radiatum are partly mediated by GluA2 (Siegel et al., 1995).

The developmentally-associated changes in GluA1 and GluA2 levels may be a function of developmentally-linked changes in the function of synaptic regulatory proteins. The membrane-associated guanylate kinases (MAGUKs) associate with the C-terminus and function as anchor proteins to stabilize AMPAr at the synapse. They also contribute to synaptic plasticity mechanisms through the insertion of AMPAr (Petralia et al., 2005; Henley and Wilkinson, 2016). The PSD-95 group, including PSD-95, PSD-93, SAP102, and SAP97, are the most widely studied MAGUKs, of which PSD-95 and SAP97 interact with and regulate subunit insertion at the postsynaptic density (Iwakura et al., 2001; Schnell et al., 2002; Tomita et al., 2003; Petralia et al., 2005; Henley and Wilkinson, 2016). Their developmental timeline has been shown to correlate with that of the GluA1 and GluA2 levels; PSD-95 and SAP97 levels first emerge around P10 and significantly increase up to P35 (Sans et al., 2000; Petralia et al., 2005; Bian et al., 2012; Pandey et al., 2015). Transmembrane AMPA receptor regulatory proteins (TARPs), such as stargazin, also function to modify and regulate AMPAr density at the synapse throughout development by facilitating the binding of AMPA with MAGUKs (Petralia et al., 2005). They, too, show a developmental pattern that parallels GluA1 and GluA2; Blair et al. (2013) reported a significant increase in TARP from P17-19 to P22-24.

The development patterns of GluA1 and GluA2 coincide with the emergence of spatial processing capabilities, with multiple reports citing improvements on hippocampal-dependent memory tasks during this period (Rudy et al., 1987; Langston et al., 2010; Raineki et al., 2010; Akers et al., 2012; Comba et al., 2015). These improved functions may be attributable to increased GluA1 and GluA2 levels given their contributions to LTP and synaptic plasticity. AMPAr insertion into the synapse during early development is initially low, resulting in synapses possessing mainly NMDAr, referred to as “silent synapses” (Rumpel et al., 1998; Liao et al., 1999; McHail and Dumas, 2015). Within the first two postnatal weeks, AMPAr insertion increases, reducing the number of silent synapses, and facilitating LTP expression and synaptic strengthening, largely mediated by the phosphorylation of GluA1, leading to more GluA1 at the synapse (Isaac et al., 1995; Durand et al., 1996; Liao et al., 1999). This cycle of LTP expression/GluA1 insertion, coupled with the increased neurogenesis and synaptogenesis observed during this time of hippocampal remodeling, would likely saturate the synapse with GluA1 and decrease the threshold of LTP expression, increasing the strength and efficacy of the synapse, and resulting in the reported improvements in cognitive function.

Although GluA2 levels may not be directly involved with LTP expression, its regulatory role likely influences improvements in spatial processing seen during this time. GluA2-lacking AMPAr are largely Ca^+2^-permeable and are present earlier in development. While an influx of Ca^+2^ into the cell is crucial for LTP and synaptic plasticity, it also renders the cell vulnerable to excitotoxicity and can oversaturate the synapse, leading to a net cessation of LTP expression and memory impairments if left unregulated (Barnes et al., 1994; Shimshek et al., 2006). Chronic neuronal excitation also leads to a decrease in excitatory synaptic transmission, known as synaptic downscaling (Siddoway et al., 2014). As such, an increase in GluA2 levels may help regulate Ca^+2^ influx via synaptic scaling, preventing oversaturation of LTP that may occur during this developmental period as a result of increased GluA1 levels. This would allow for a preservation of encoded information, while allowing for the continued strengthening of the synapse, and the reported improvements in cognitive function.

The pattern of GluA1 and GluA2 development within the ACC is similar to what has been found in previous investigations showing stagnated levels within the neocortex during this time (Pellegrini-Giampietro et al., 1991, 1992b; Orlandi et al., 2011). Despite this lack of developmentally-linked changes in ACC GluA1 and GluA2 levels during the P18 – P50 period, GluA1 and GluA2 levels do show changes from P5 to P20, suggesting that the subunits within the ACC undergo changes earlier in postnatal development relative to the hippocampus (Pellegrini-Giampietro et al., 1991, 1992b; Orlandi et al., 2011). Additionally, by P21, the cortical layers in the dorsal anterior cingulate have fully matured and the number of medial prefrontal cortex (mPFC) neurons have stabilized (van Eden and Uylings, 1985; Juraska and Willing, 2017). Despite this, the mPFC continues to show developmental changes including an increase in synaptic boutons from P35 to 45 (Drzewiecki et al., 2016), a spike in basal an apical dendritic spine density at P35 (Juraska and Willing, 2017), and a maturation of pre- and post-synaptic makers, like PSD-95 and synaptophysin, by P50 (Pinto et al., 2013; Testen et al., 2019). As the timeline used in this investigation falls between the timeline of this bimodal developmental pattern, the changes in GluA1 and GluA2 may not be detected at this time.

## 5 Conclusion

During preadolescence, the hippocampus undergoes developmentally-linked remodeling (P18 – P24; as previously reported) that is associated with changes in the expression of GluA1 and GluA2 levels (P18 – P24; reported currently). The developmental maturation of the rodent hippocampus during the preadolescent period is reflected in the emergence of spatial/allocentric memory processing (P20 – P24; Holahan et al., 2019) indicating near-simultaneous developmental changes in connectivity, synaptic excitation and memory function. We also examined whether changes in GluA1 and GluA2 expression in the ACC would peak during the preadolescent period. Developmentally-linked changes in connectivity patterns in the ACC occur during the preadolescent period (P22 – P36; previously reported), but there does not appear to be a corresponding change in GluA1 or GluA2 expression (no changes from P18 – P30; current report). There also does not appear to be a clear link between the developmental changes in the ACC and the memory functions associated with the ACC reported to emerge around P20 (Guskjolen et al., 2017). In essence, while the hippcampus shows rapid, simultaneous changes in structure and function during preadolescence, the developmental changes in the ACC appear to be more protracted and sequential and may continue well into the adolescent period.

## Conflicts of interest

The authors declare that the research was conducted in the absence of any commercial or financial relationships that could be construed as a potential conflict of interest.

## CRediT authorship contribution statement

**Nikolaos Tzakis:** Validation, Formal analysis, Investigation, Writing - original draft, Writing - review & editing, Visualization, Project administration. **Matthew R. Holahan:** Conceptualization, Methodology, Validation, Formal analysis, Investigation, Resources, Writing - original draft, Writing - review & editing, Visualization, Supervision, Project administration, Funding acquisition.
