# Supplementary figures and images for: Investigation of GluA1 and GluA2 AMPA receptor subtype distribution in the hippocampus and anterior cingulate cortex of Long Evans rats during development
Source: IBRO Rep. 2020 Apr 3;8:91–100. doi: 10.1016/j.ibror.2020.03.003 (PMC7152689; doi:10.1016/j.ibror.2020.03.003)

**Supplementary Figure 1.** Western blot showing target protein for (A) GluA1 and (B) GluA2.


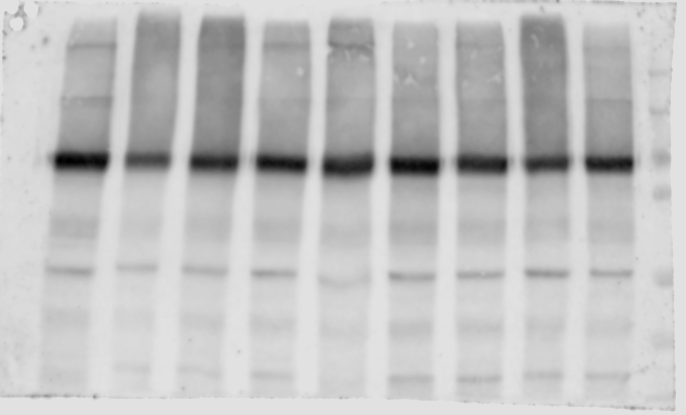

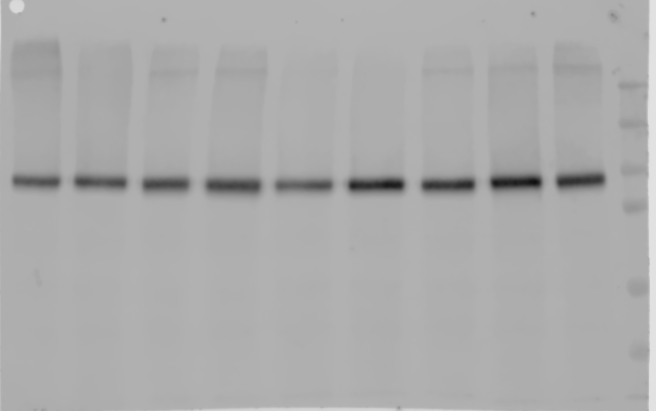


**A.**

**B.**

**~100 kDa**

**~100 kDa**

Supplement: Supplementary file 1 [file mmc1.docx]
